# Green Methods to Recover Bioactive Compounds from Food Industry Waste: A Sustainable Practice from the Perspective of the Circular Economy

**DOI:** 10.3390/molecules29112682

**Published:** 2024-06-05

**Authors:** Vincenzo Roselli, Gianluca Pugliese, Rosalba Leuci, Leonardo Brunetti, Lucia Gambacorta, Vincenzo Tufarelli, Luca Piemontese

**Affiliations:** 1 Department of Pharmacy-Pharmaceutical Science, University of Bari Aldo Moro, Campus E. Quagliariello, Via E. Orabona 4, 70126 Bari, Italy; 2 Department of Precision and Regenerative Medicine and Jonian Area (DiMePRe-J), Section of Veterinary Science and Animal Production, University of Bari Aldo Moro, 70010 Valenzano, Italy; 3 Institute of Science of Food Production (ISPA), Research National Council (CNR), Via Amendola 122/O, 70126 Bari, Italy

**Keywords:** agri-food by-products, natural bioactive compounds, extraction methodologies, green impacts and sustainability, deep eutectic solvents, green chemistry

## Abstract

The worrying and constant increase in the quantities of food and beverage industry by-products and wastes is one of the main factors contributing to global environmental pollution. Since this is a direct consequence of continuous population growth, it is imperative to reduce waste production and keep it under control. Re-purposing agro-industrial wastes, giving them new life and new directions of use, is a good first step in this direction, and, in global food production, vegetables and fruits account for a significant percentage. In this paper, brewery waste, cocoa bean shells, banana and citrus peels and pineapple wastes are examined. These are sources of bioactive molecules such as polyphenols, whose regular intake in the human diet is related to the prevention of various diseases linked to oxidative stress. In order to recover such bioactive compounds using more sustainable methods than conventional extraction, innovative solutions have been evaluated in the past decades. Of particular interest is the use of deep eutectic solvents (DESs) and compressed solvents, associated with green techniques such as microwave-assisted extraction (MAE), ultrasonic-assisted extraction (UAE), pressurized liquid extraction (PLE) and pulsed-electric-field-assisted extraction (PEF). These novel techniques are gaining importance because, in most cases, they allow for optimizing the extraction yield, quality, costs and time.

## 1. Introduction

Recent studies have demonstrated that continuous population growth is leading to a significant increase in total food demand (the current forecast data indicate it will grow by 26% between 2010 and 2050) [1]. This trend could lead to a significant increase in food by-products and waste, with important and non-negligible environmental impacts. In fact, the food and beverage industry’s global production activity is currently estimated to contribute 30% of the whole environmental impact of human activity, and this worrying percentage increases to more than 50% if we consider eutrophication, a process by which the concentration of oxygen in water systems, such as estuaries and lakes, increases as a consequence of enhanced plant growth caused by an excess of dissolved nutrients [2].

Food waste, which has gained increasing attention in the last decades, can be defined as “any edible or inedible food loss from the food-supply chain” [3]. Fruit-based and plant-based wastes are particularly important, considering that ~10–60% of the total weight of fresh vegetables and fruit, produced in massive quantities, is inevitably discarded [4], with around half of the harvest lost every year (FAO, 2011) [5]. Wastes also arise due to the presence of inedible components (mostly outer layers, pomace, seeds and peels), which are usually discarded and account for different percentages in weight depending on the source (from 12% for apples up to 46% for pineapples) [5,6]. These inedible parts can still play an important role as “secondary raw materials” thanks to their content in bioactive compounds such as proteins, dietary fibers, polysaccharides and phytochemicals, including secondary metabolites [5,7,8,9,10]. Phytochemicals are a large group of low-molecular-weight organic substances, including phenols/polyphenols, terpenoids and alkaloids. They can be defined as plant-derived compounds with beneficial effects on human or animal health [8,10,11]. Phenolic compounds are particularly attractive, given their involvement in important physiological mechanisms, along with their antioxidant, anti-inflammatory, antimicrobial, anti-allergenic, antithrombotic and cardioprotective activities [5,8]. Many epidemiological studies associate the constant intake of polyphenol-rich foods or beverages with the prevention of several chronic diseases [8,10]. This is because polyphenols, together with vitamin E, vitamin C and carotenoids, act as reducing agents, protecting the body’s tissues against oxidative-stress-based pathologies [12].

The aim of this review is to change the current point of view on five different industrial waste products, and to highlight their potential as resources. We consider both “conventional” waste products, whose nutraceutical and other applications have been well-known for many years (banana, pineapple waste and citrus peels), and “unconventional” ones (cocoa bean shells and brewers’ spent grain), which have been studied only in the past two decades. Although the presence of inedible compounds or toxic contaminants places a limit on the usage of these waste products [13], it can be overcome by extracting bioactive compounds, which also serves to concentrate these substances [14].

Extraction can be performed using various technologies and solvents, but to follow a virtuous cycle of sustainable use of food waste, it must be designed according to the principles of green chemistry [14]. The main green solvents that will be reported in addition to the conventional ones are DESs and compressed solvents (e.g., supercritical liquids, pressurized water). The extraction process can be assisted by other green techniques, such as MAE, UAE, PLE and PEF. Their application can optimize yields and reduce the extraction time, making the whole procedure more energy efficient [14].

## 2. Alternative Methods for Green Extraction of Bioactive Compounds from Vegetable/Fruit-Based Waste

### 2.1. New Sustainable and Innovative Extraction Techniques

**Figure 1:**
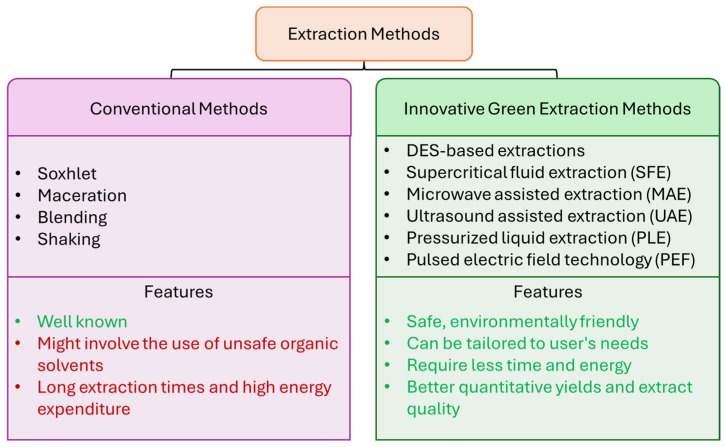
Traditional (conventional) and innovative extraction methods.

Usually, traditional extraction techniques are very time-consuming and require high amounts of solvents and energy for heating and/or stirring. These problems are solved, completely or partially, using novel environmentally friendly extraction methods (Figure 1), which can be used to optimize the whole extraction process in terms of quantitative yield, extract quality and extraction time.

They can be summarized as follows:

- *DES-based extraction.* DESs are considered green solvents thanks to their non-flammability, chemical and thermal stability, low volatility and toxicity [15]. In some cases, their use can also lead to higher extraction yields and extract quality [15]. Among the most studied DESs, choline-chloride-based DESs are of great interest, since it is possible to modify their physicochemical properties by varying the hydrogen bond donor. Thus, their viscosity, pH and polarity can be tailored to their application [16].
- *Supercritical fluid extraction (SCFE)*. SCFE is a technique that uses a fluid at temperatures and pressures above its critical point [17]. Under these conditions, the fluid exhibits properties that are between those of a liquid and gas, so that higher diffusivity and lower surface tension, density and viscosity are shown compared to conventional solvents [17].
- *Microwave-assisted extraction (MAE)*. This technique consists of internally and externally heating the samples without using any thermal gradient [18,19]. Since microwaves are strongly absorbed by polar molecules, such as ionic solutions, the consequent internal superheating of water molecules of a matrix leads to cellular disruption, with enhanced extraction of compounds of interest from the matrix itself [18,20]. However, microwave power is usually kept under 500 W in order to avoid a considerable decrease in the total flavonoid content and scavenging activity caused by overheating and degradation of antioxidant molecules [18].
- *Ultrasound-assisted extraction (UAE)*. Ultrasound energy and solvents are used to recover target compounds from a wide range of plant matrices [21]. Ultrasound waves induce the formation of cavitation areas in liquids, leading to increased displacement of the molecules from their positions [22]. Ultrasound action results in increased solvent permeability and diffusivity in matrices [23]. It also helps to increase the volume of the plant tissue matrix (swelling index), which is important for the diffusion of solutes during extraction processes [22,24]. A UAE system can be defined as a cost-effective, efficient, environmental friendly and easy-to-use procedure [25].
- *Pressurized liquid extraction (PLE)*. Extraction occurs at a temperature between the boiling point and the critical point of the solvent (usually water), improving extraction kinetics [18,26]. The operating pressure must be high enough to keep the solvent in its liquid state [18,26].
- *Pulsed electric field (PEF) technology*. This is a promising short-duration extraction technique, in which high-intensity pulsed electric fields are applied [27], producing electroporation and thus increasing the permeability of cell membranes, leading to higher extraction yields [28,29].

### 2.2. Plant Waste Matrices under Study

Despite their cultivation in tropical and sub-tropical areas [30], bananas are among the most popular fruit worldwide. Banana peels have potential utility for oil sorption [31], production of biomass energy [32] and biofuel (methane and bioethanol) [33], bioadsorbent for dyes [34] and synthesis of biodegradable plastic material [35]. Thus, beyond its nutraceutical value, this waste material has multiple applications—mostly due to its high content of polyphenols—on which we will focus later.

Citrus peel’s chemical composition and nutraceutical potential are very well-known, and this waste material is mostly reused for the production of essential oils, for the recovery of antioxidant compounds and for the production of biofilm for food packaging [36].

Pineapple is a complex fruit, and both its edible and non-edible parts are sources of many micronutrients and valuable compounds. Nonetheless, there are many discarded parts (core, peel, seeds, stem, crown, leaves) and those account for a significant percentage of the total fruit weight [37]; thus, it is imperative to find additional uses for them. In addition to its nutraceutical value, pineapple waste has been recognized as a good bioadsorbent material, easily regenerable and suitable for water treatment [38], as well as the production of biogas [39] and nanocellulose-based material [40].

Brewers’ spent grain is the main by-product of beer production [41]. Its usage as a source of polyphenols has been explored in depth in recent times, and it may have many other meaningful features that are not yet known. Brewers’ spent grain has been proposed as a starting material to produce value-added food, mostly bakery products [42], or as a source of components (cellulose, xylitol, lignin and arabinoxylan) to produce sustainable food packaging [43].

Finally, the large diffusion of and demand for cocoa and chocolate-based food, and the fact that cocoa production has a very significant environmental impact [44], make it necessary to try and give new life to the many by-products that are inevitably produced by this industry. One of these by-products is the cocoa bean shell, which has several potential applications as a starting material for biopolymer production, an absorbent of contaminants in wastewater and a corrosion-inhibitor agent, along with offering a potential source for the formulation of functional foods and beverages [45].

A series of papers on different extraction methodologies applied to the above-mentioned by-products are reviewed in the next sub-sections and summarized in Table 1.

#### 2.2.1. Banana Peels

The significant nutritional content of banana and its popularity [46] make its peel, which makes up about 35% of the total fruit weight and which is produced in a quantity of around 36 million tons annually [47,48], a precious material. In fact, more than forty phenolic compounds, such as flavonols, flavan-3-ols, hydroxycinnamic acids and catecholamines, have been detected in the raw material [48].

As already observed in other matrices, for banana peel, conventional solid–liquid extraction techniques, such as Soxhlet extraction and maceration, are gradually being replaced by innovative ones (e.g., MAE, UAE, subcritical water extraction and avoiding too-high temperatures that could affect thermolabile bioactive compounds). Traditional Soxhlet extraction leads to higher extraction yields but a worse extract quality than subcritical water extraction. Microwave-assisted extraction (MAE), instead, can be carried out with a significantly shorter extraction time and at a lower temperature than Soxhlet extraction, with an enhanced extract quality [49]. The use of ultrasound-assisted extraction (UAE) also represents an improvement on Soxhlet extraction in multiple regards (energy, time and quality), but can lead to the degradation of phenolic acids [49].

In 2022, an interesting study was published highlighting the difference between sonication and maceration techniques in terms of polyphenol recovery from banana peel. UAE showed better results in terms of extraction yield (=weight of extract/weight of sample × 100, 13.48% at 45 °C) in a very short time (1 h). The authors also observed that the best solvent to obtain and preserve bioactive substances was 50% ethanol in water, which reached a total polyphenolic content (TPC) of 31.46 mg GAE/g with a sample/solvent ratio of 1:20 [50].

Bello et al. [51], instead, used supercritical CO_2_ as the extraction solvent for a milled banana peel sample by adding a 5% volume of ethanol as a co-solvent with a flow rate of 9.8 g/min). In the best operating conditions (80 °C, 25 MPa, 2.5 h), a yield of 1.58% was achieved and the extract was found to contain a significant quantity of important bioactive compounds such as gallic acid, quercetin and beta-carotene [51].

Encouraging results were also obtained in a recent investigation [52] using homogenizer-assisted extraction (HAE). In this case, milled banana peel was extracted using an ethanol:water solution with a sample–solvent ratio of 2.5–7.5% (*w*/*v*; best result: 7.5%) and a concentration of 20–70% (*v*/*v*; best result: 54%). The extraction process, in which Ultra-Turrax Homogenizer set at 11,000 rpm was used, took only thirty seconds, and the total phenolic content (2.44 g GAE/100 g dw, dry weight) as well as the antioxidant activity (estimated with three different assays) of the extract were high [52].

Aziz et al. [53] performed the extraction of phenolic compounds from various plant waste materials, including banana peel, by using different types of natural deep eutectic solvents (NADESs; e.g., choline chloride, ChCl/glucose, ChCl/lactic acid, ChCl/ascorbic acid, glucose/lactic acid, glucose/ascorbic acid, sucrose/ascorbic acid, sucrose/lactic acid), each one at the molar ratios of 1:1, 1:2 and 2:1, at room temperature and in a shaker set at 100 rpm for 20 min. The most efficient combination was choline chloride/ascorbic acid at 1:2 (mol/mol) [53].

Hendrawan et al. [54] demonstrated that PEF, rather than a standalone extracting method, is a useful preliminary procedure to increase the phenolic yield recovered from Kepok banana peel. In fact, both the total phenolic content and antioxidant activity were higher than in the control sample not pre-treated with PEF (1.090 ± 0.165 mg GAE/g dry extract). In their study, the electric field strength of 4 kV/cm for 2 min led to the highest value of total phenol content (1.664 ± 0.226 mg GAE/mg dry extract), while considering antioxidant activity, the highest value was reached using the same electric field strength for 4 min (IC_50_ = 13.086 ± 4.547 mg/mL; DPPH assay) [54].

#### 2.2.2. Citrus Peels

As already mentioned, citrus fruits, considering the different species distributed throughout much of the world, are a valuable source of bioactive phytochemicals [55]. Despite this, the potential of citrus peel is usually undervalued and neglected, wasting, during citrus juice processing, thousands of tons of this precious material [55,56], including carotenoids and ascorbic acid but also flavanones, polymethoxylated flavones and phenolic acids [55,57,58,59,60,61].

The main innovative methods that have been employed in recent years to extract these antioxidant compounds from citrus peels are green solvents, supercritical CO_2_, PEF-assisted extraction, pressurized fluid extraction, high-pressure-assisted extraction, MAE, UAE, anaerobic digestion and enzyme-assisted extraction [62,63,64,65].

In order to extract flavonoids from citrus peel, recently, a series of tailor-made DESs were tested [66] using, as a hydrogen bond donor, choline chloride, and, as hydrogen bond acceptors, carboxylic acids, different amides, sugars and alcohols. The best choice to perform the extraction was found to be a DES consisting of a ternary system composed of choline chloride, levulinic acid and methyl urea (ChCl/LeA/MU 1:1.2:0.8 mol/mol, with 20% of water) [66]. It was observed that this DES, under optimized conditions (solid–liquid ratio of 1:50 *w*/*v* at 50 °C for 25 min), provided a yield of 65.82 mg/g of total flavonoids, a value that confirmed the effectiveness of this DES over the most useful conventional solvent tested (using methanol, 53.08 mg/g of total flavonoids was obtained) [66].

Among the innovative green methods described above, supercritical CO_2_ extraction seems to be the most eligible for extracting essential oils and lipophilic compounds, but it is less efficient for phenolic compounds [63]. This was recently confirmed in a study conducted by Šafranko et al. on mandarin peel [67] using a two-step green extraction technique consisting of supercritical CO_2_ treatment and then subcritical water extraction (SWE) [67]. The first extraction was carried out under a CO_2_ mass flow rate of 2 Kg/h, at 40 °C at 100 or 300 bar for 1.5 h. After this treatment, limonene was the major volatile biomolecule extracted at both pressures used, with a yield of around 13% at 100 bar and 31% at 300 bar, followed by α-farnesene, linoleic and hexadecanoic acids. The following SWE extraction was performed in order to also recover phenolic compounds, the most abundant of which were hesperidin (extracted in a quantity of 15.05 mg/g at 153 °C, 15 min and 30 mL/g molar ratio), rutin (3.79 mg/g obtained at 168 °C, 10 min and 30 mL/g), narirutin (5.05 mg/g at 140 °C, 15 min and 29 mL/g) and chlorogenic acid (68.76 mg/g at 219 °C, 9 min and 30 mL/g) [67]. On the other hand, El Kantar et al. demonstrated that PEF treatment (at 10 kV/cm), using a 50% ethanol aqueous solution at 50 °C for 1 h, improves polyphenol yields (22 mg/g DM) compared to conventional extraction (12 mg/g DM), using the same solvent, starting from the skins of different citrus fruits, particularly from orange peel [62].

Considering lime peel, an interesting extraction process was conducted through UAE and MAE [68]. The optimized protocol required, respectively, the use of 55% ethanol solution and 38% of the ultrasonic amplitude for 4 min, and 55% ethanol solution and 140 W of microwave power for 45 s, repeating the extraction procedure 8 times. In the described conditions, UAE was more efficient and fast than MAE to extract the total phenolics (with total phenolic contents of 53 ± 1 mg GAE/g and 54 ± 1 mg GAE/g, respectively), leading to extracts with comparable high antioxidant activities (18 ± 0.2 μM Trolox/g and 19 ± 0.4 μM Trolox/g, respectively, for the DPPH assay) [68].

A relatively recent trial on the use of the UAE technique for Kinnow mandarin peels demonstrated its higher efficiency than maceration, with a recovery of eleven phenolic compounds (including four flavonoids and five phenolic acids, with hesperidin and ferulic acid as the most abundant ones) [69]. The best solvent used was 80% methanol-water solution (together with methanol, ethanol was also tested at three concentrations: 50%, 80% and 100%) under the optimal conditions, consisting of a sample/solvent ratio of 1:20, at 45 °C, for 60 min, in an ultrasound bath at a frequency of 35 kHz. Under these operating parameters, a total polyphenol content of 32.48 mgGAE/g was reached, and a scavenging activity of 72.83 ± 0.65% was obtained (evaluated using the DPPH assay) [69]. Montero-Calderon et al. also used UAE (400 W, 50% ethanol, 30 min) on orange peel, obtaining significant values of total carotenoid content (0.63 ± 0.01 mg β-carotene/100 g), vitamin C (53.78 ± 3.36 mg AA/100 g) and phenolic concentration (105.96 mg GAE/100 g), with an antioxidant capacity, expressed as the oxygen radical absorbance capacity, of 27.08 mM Trolox Equivalent (TE) [70].

It has also been recently demonstrated that PLE achieves higher extraction yields of glycosylated flavonoids from orange peel—and PLE also requires a shorter extraction time and lower temperatures—not only compared to Soxhlet conventional extraction but also to UAE [71]. In detail, a preliminary supercritical CO_2_ treatment (40 °C, 35 MPa) was able to extract an essential oil highly enriched in α-terpineol. Then, PLE was conducted for the optimized time of 20 min, at 45–65 °C and with the pressure set at 10 MPa using ethanol in water at three different concentrations (50%, 75% and 99.5 *v*/*v*) [71]. The highest phenolic compounds’ recovery (14.9 ± 0.7 and 15.9 ± 0.2 mg GAE/g dry peel for the sample untreated with supercritical CO_2_ and the treated sample, with scavenging activities of 4.6 ± 0.3 and 5.1 ± 0.3 mg TE/g dry peel, respectively) was reached at 65 °C and with 75% ethanol [71].

A study conducted by Casquete et al. [72] confirmed that high-pressure processing (HPE), used to extract phytochemicals from orange and lime peels (a pressure of 300 MPa was applied for 10 min on orange peel, and 500 MPa for 3 min on lemon peel) with an 80% ethanol-water solution, leads to a higher total phenolic compound content (400 mg GAE/100 g and 344.53 mg GAE/100 g fresh peel extracts, respectively) than control samples, resulting in enhanced antioxidant effects (136.85 mg Trolox/100 g and 149.41 mg Trolox/100 g fresh peel extracts, respectively) [72].

Finally, Li et al. reported that, with regards to the enzyme-assisted extraction of phenolic compounds from citrus peel, Celluzyme MX gave the highest total phenolic content with a recovery of up to 65.5% (50 °C, 3 h, 2:16 *m*/*v* frozen citrus peel powder: enzyme solution) [73].

#### 2.2.3. Pineapple Wastes

Globally, pineapple is one of most highly produced tropical fruits, and more than 10,000 km^2^ of soil is dedicated to its cultivation [74,75]. The derived wastes (peel, core, crown, stem and leaves), which are generally eliminated in the course of processing, account for about 50% (*w*/*w*) of the total pineapple weight [37]. These parts are a rich potential source of bioactive polyphenols such as myricetin, tannic acid, salicylic acid, trans-cinnamic acid, *p*-coumaric acid [37] and bromelain, a mixture of different enzymes (including thiol endopeptidases, protease inhibitors and others) with confirmed therapeutic benefits in many studies [76].

Microwave-assisted extraction has been recently used in a published study as a more efficient alternative to conventional Soxhlet and maceration techniques [77]. The best conditions to reach the highest yield of phenolic compounds (207.72 mg GAE/g dw) and the highest antioxidant activity (13.2 mg/mL of DPPH value) consisted of operating at 60 °C with 750 W of microwave power for 20 min, using 50% ethanol in deionized water as the solvent [77]. The standard Soxhlet extraction obtained a lower total phenolic content (28.78 mg GAE/g dw) as well as antioxidant activity (2.78 mg/L) [77].

Instead of ethanol, the use of two DESs (choline chloride–glycerol 1:2 with 10% water and choline chloride–malic acid 1:1.5 with 50% water) in MAE, with a 2.55 MHz operating frequency, has been experimented with as an alternative method for extracting phenolic compounds from pineapple peel [78]. Choline chloride–glycerol was found to be the best DES for this purpose, and pre-treating the solid sample at the optimum drying temperature of 67 °C and using a liquid/solid ratio of 60.5 mL/g for 87 s were found to be the optimized parameters that permitted extracts to be obtained with a high concentration of total phenolic compounds (35.95 mg GAE/gdw, compared with 7.98 mg eq GAE/gdw obtained by using traditional solvents) and high antioxidant activity (28,630 μMeq Trolox/g dw) assayed with the DPPH assay [78]. The application of Box Behnken design optimization to MAE was also tested in a trial [79] in which three parameters were varied and evaluated: the substrate ratio (the interval 10:1–20:1 mL/g was investigated) using ethanol/water 50:50 as the solvent, microwave power (from 300 W to 600 W) and extraction time (from 40 min to 50 min) [79]. Optimization processes led 20:1 mL/g, 600 W and 40 min to be determined as the optimal conditions, obtaining a total phenolic content of 14.88 mg GAE/gdw, a total flavonoid content of 12.925 mg QE/g dw and a total tannin content of 371.25 mg TAE/g dw [79].

Paz-Arteaga et al. [80] used UAE to extract polyphenols from pineapple waste but, in order to increase their release, they preliminarily fermented the samples with microfungi (*Aspergillus niger*) for 32 h (the optimized process required 86 mg of fermented material per each mL of solvent, 26% ethanol in water and two cycles of sonication using a vortex at 9000 rpm for 1 min with a frequency of 40 kHz at 37 °C) [80]. The final extracts showed significant antimicrobial activity against *L. monocytogenes* and *S. aureus*, dangerous bacteria that contaminate food [80].

Another paper reported UAE combined again with preliminary solid-state fermentation (SSF) on some samples of Golden pineapple peels, using an ultrasonic bath at 35 kHz and varying the ethanol concentration, time and temperature [81]. Under optimal conditions (58% ethanol, 62 °C and 30 min), the extracts had a total phenolic content of 866.26 mg GAE/g and an activity (DPPH method) of 63.53 ± 2.02% [81]. UAE was also used in the study of Yahya et al., using 50% ethanol as the solvent at 30 °C [82]. The highest total phenolic (1078.68 ± 1.32 mg GAE/g dw) and flavonoid (1276.64 ± 5.92 mg QE/g DW) contents were achieved after a sonication time of 5.92 min (acting at 100% sonication amplitude: 130 W) [82]. On the other hand, Soxhlet extraction (7.98 mg GAE/g dw) and maceration (51.10 ± 0.20 mg GAE/g dw) gave a considerably lower total phenolic content [82,83,84].

Further experiments have been reported by Zampar et al. [85]. In particular, different solvents (ethanol, water and 1 mol/L HCl solution) were mixed in various proportions, in order to obtain the most effective solvent for UAE in terms of polyphenol extraction capability [85]. The effects of temperature (30–60 °C), time (5–45 min) and ultrasonic power (0–100 W) were investigated, leading the authors to conclude that the maximum total phenolic content (405.06 mg GAE/100 g) can be obtained using, as a solvent, a mixture of ethanol (0.50) and acid solution (0.50) for 30 min at 60 °C, and using an ultrasonic power of 50 W [85].

UAE and MAE technologies have also been employed for bromelain and other bioactive peptides’ extraction by T. Mala et al. on various pineapple by-products (crown, peel and core) [86]. They obtained extracts with a proteolytic activity of 196.46  ±  3.29 U/mL for UAE and 154.08 ± 1.49 U/mL for MAE by using distilled water as the solvent [86]. The best operating parameters for UAE consisted of an ultrasonic amplitude of 99.6% and a water/solid material ratio of 20.96 mL/g for 26.83 min. For MAE, the optimal parameters were 100 W of microwave irradiation power and a solid material/distilled water ratio of 1:8 g/mL for 8.99 min [86]. Ultrasound-assisted liquid-phase microextraction (UA-LPME) was applied on pineapple leaves, peels and stems, combined with the use the use of NADESs, as extracting solvents by Balaraman et al. [87]. The best NADES was found to be tetrabutyl ammonium chloride:imidazole:glycerol (1:1:1), with a liquid/solid ratio of 25 mL/g, at 45 °C for 17.5 min of irradiation, obtaining an high yield (87%) of bromelain that was purified by final gel filtration chromatography [87].

Autohydrolysis, in which only water is used as the extraction solvent, seems to be an interesting method for the sustainable extraction of high-value-added molecules. Sepùlveda et al. [88] used this technique for pineapple wastes, reporting a high polyphenol recovery (1.75 g/L) in the following optimized conditions: 30 min at 200 °C with a 1:10 *w*/*v* solid–liquid ratio [88]. The phenolic compounds that were detected were epicatechin, gallic, hydroxybenzoic, coumaric, chlorogenic and caffeic acids [88].

#### 2.2.4. Brewery Waste

Beer has a prominent place among the alcoholic beverages most consumed worldwide [89]. It has been estimated that more than 194 billion liters were produced globally in 2018, for which around 130 thousand tons of hop were necessary [90,91,92]. The large amounts of solid waste generated by the brewery industry (around 134 thousand tons in 2019 according to FEOSTAT 2022) can be divided into three types: brewers’ spent grains (BSGs), which reach quantities of around 14–20 kg for each 1 hL of brewed beer, spent brewers’ yeast (SBY) and spent hops [93,94].

Brewers’ spent grain, the most abundant solid waste generated during beer production, has an unexpectedly valuable chemical composition. Among the most represented compounds in this by-product, arabinoxylans and β-glucans are the main fibers and, along with phenolic components, such as hydroxycinnamic acids (mostly sinapic, ferulic, *p*-coumaric and caffeic acids), have great importance for human health [41,95]. Proteins are around 20% of the total dry mass [95], while lipids are only a small part, among which triglycerides are the main constituents (accounting around 67%), followed by a smaller amount (around 18%) of fatty acids [41,96]. Other micronutrients such as calcium, phosphorus and magnesium are also found in BSG [95,97], as well as traces of iron, manganese, copper and potassium [98]. The vitamins found, meanwhile, are niacin, folic acid, biotin, choline, thiamine, pantothenic acid, riboflavin and pyridoxine [97,99,100]. In addition to all these important components, resins, waxes, gums, essential oils, tannins and other cytoplasmatic components have been detected as well in numerous analyses [41,100].

An efficient MAE process to recover polyphenols—in particular, ferulic acid—from BSG has been developed by Moreira et al., simply by modulating the pH of aqueous media [101]. The results for this MAE application showed that using an extraction time of 15 min at 100 °C with a ratio of solvent (NaOH 0.75% *v*/*v*) to raw material of 20:1 under the maximum stirring speed led to a fivefold higher ferulic acid yield (1.31 ± 0.04% *w*/*w*). These results were compared to those of the conventional extraction techniques, such as mechanical stirring (methanol 70%, 30 min at 25 °C), alkaline hydrolysis (NaOH 2% *v*/*v*, 90 min at 110 °C) and Soxhlet extraction (ethanol, 4 h at the boiling point of the solvent) [101].

DESs have been proposed as an alternative to hydroalcoholic or aqueous solutions for the microwave-assisted extraction of phenolic compounds from BSG [102]. It has been reported that ChCl:glycerol (with a molar ratio of 1:2) is the most efficient DES for this purpose (ChCl/lactic acid 1:2 mol/mol, ChCl/ethylene glycol 1:2 mol/mol and ChCl/1,2-propanediol 1:2 mol/mol were tested, as well) compared to the conventional solvent, methanol (80% in water, *v*/*v*; 1.2 mg GA/g BSG). The optimal conditions found were 13.30 min of extraction time, 100 °C and 37.46% (*v*/*v*) water used in the DES, generating a liquid extract with the highest antioxidant power (2.89 mg GA/g BSG) [102].

Polyphenols and flavonoids have also been extracted from BSG by Spinelli et al. [103] using supercritical CO_2_ (the solvent was composed of CO_2_ + 60% ethanol *v*/*v*). The optimal temperature was 40 °C, with a pressure of 35 MPa and with a CO_2_ flow rate equal to 2 L/min. This treatment, which took 240 min, led to higher phenolic and flavonoid contents (30% and 50%, respectively) and a better antioxidant activity than the control samples. Importantly, this can be followed by a microencapsulation process, preserving the stability of polyphenols and flavonoids and covering their bad taste [103].

PLE has recently (2021) been tested in a new method that employs ethanol in water with a percentage from 0% to 100%, a temperature that ranged from 25 to 155 °C and an extraction time of from 3 to 17 min [104]. Different extracts were obtained, and the highest content of phenolic compounds (1.72 ± 0.07 g GAE/100 g BSG) (coumaric, transferulic and p-hydroxybenzoic acids were present) was reached under the optimized conditions of 155 °C, 35% ethanol and a 17 min extraction time [104].

Martín-García et al. [105] applied PEF treatment as a preliminary procedure for conventional solid–liquid extraction in order to improve phenolic recovery using different parameters of time (5, 10 and 15 s), electric field strength (0.5, 1.5 and 2.5 kV/cm) and frequency (0.05, 0.1 and 0.15 kHz) and employing, as the extracting solvent, ethanol/water (4:1 *v*/*v*) [105]. The results showed that, when using PEF treatment before the extraction and the optimal operating conditions (14.5 s, electric field strength of 2.5 kV/cm and frequency of 0.05 kHz), the extraction yield and the antioxidant activity improved compared to the non-pre-treated procedure [105].

Alonso-Riaño et al. [106] demonstrated that water UAE (47 °C; solvent volume-to-dry BSG mass ratio (*v*/*w*) of 21.7 mL:g BSG dry; and 30 min of sonication) is much more efficient in the extraction of polyphenols than conventional extraction. UAE results in a significant improvement of final extraction yields, reaching 55% for non-ground BSG and 30% for ground BSG (the productivity was found to be 0.109 mg GAE/g BSG dry·min for UAE and 0.0078 mg GAE/g BSG dry·min for the conventional extraction) [106]. However, UAE shows a lower efficiency than other hydrolytic extraction methods such as basic hydrolysis, owing to the impossibility of extracting phenolic compounds linked through ester bonds to the cell wall. Mussatto et al. [107] used basic hydrolysis to recover ferulic and *p*-coumaric acids, concluding that the optimal conditions for this with a 1:20 (*w*/*w*) solid:liquid ratio were a 2% NaOH concentration, 90 min and 120 °C, with final quantities extracted of 145.3 mg/L and 138.8 mg/L, respectively [107].

A revalorization method of BSG, proposed by Wagner et al. [43], is the so-called “acid autohydrolytic saccharification”, where enzymatic treatment works as a preliminary procedure of acid autohydrolysis. During this process, the release of polyphenols from BSG is thought to occur after the saccharification of structural polymers [108].

Finally, Gandolpho et al. made use of UAE to extract phenolic compounds from hot trub (the second main solid waste after BSG) [109]. The extraction’s optimal conditions consist of using 58% ethanol in water, a solid–liquid ratio of 1:32 (*w*/*v*) and 36 °C for 30 min, and this protocol resulted in a total phenolic content of 7.23 mg GAE/g [109].

#### 2.2.5. Cocoa Bean Shells

Around 23 thousand tons of cocoa are produced every year in Ecuador (PROECUADOR, 2013) [110] to support the demand for chocolate and cocoa-derivative foods (e.g., cocoa paste, powder, butter and liquor) [111]. Cocoa bean shells (CBSs) are one of the main by-products of this production process, and their high nutritional value allows them to be further used in the food industry, as well as in the cosmetic, pharmaceutical and agricultural industries [112]. The CBS is a precious source of valuable nutrients such as phenolic compounds (catechin and epicatechin, flavonols and procyanidins), dietary fibers, methylxanthines (mainly theobromine and caffeine) and vitamin D, which, during the fermentation process, move from the bean to the cocoa shell [113,114,115,116,117].

Mazzutti et al. [118] integrated two green techniques by first using supercritical CO_2_ (99.9%) to perform an initial defatting process (20 MPa, 40 °C, 20 min), followed by pressurized liquid extraction with 99.8% CO_2_ (10 MPa, 70 °C, 20 min), obtaining both a higher total phenolic content (from 35 to 51 mg GAE/g) and antioxidant activity (EC_50_ values from 115 to 177 μg·mL^−1^, DPPH assay) compared to non-integrated methods [118].

In a study by Okiyama et al. [119], PLE was tested using absolute ethanol for flavanoid extraction. The evaluated temperatures were 60, 75 and 90 °C, the extracting time was from 5 to 50 min, the mass ratio (solid:solvent) was kept constant at 1:3 and the system pressure was fixed at 10.35 MPa [119]. The flavanol extraction yield increased by increasing the temperature and extraction time but, as a result of doing so, the procyanidin B2 decreased gradually [119].

Jokić et al. [120] used subcritical water extraction (SWE) and noted that the total phenolic content (catechin and epicatechin, gallic and chlorogenic acids were detected) changed significantly with the extraction temperature, from 27.26 mg GAE/g (120 °C, 75 min, 20 mL/g) to 130.33 mg GAE/g (220 °C, 75 min, 20 mL/g), and that the antioxidant activity changed from 19.20% DPPH scavenging (120 °C, 75 min, 20 mL/g) to 91.69% DPPH scavenging (220 °C, 75 min, 20 mL/g) [120]. Methylxanthines such as theobromine, theophylline and caffeine were also detected in the extractions performed from 120 °C to 170 °C. Beyond 170 °C, it was found that the theobromine content started to decrease [120].

MAE showed promising results when various choline-chloride-based DESs (ChCl/lactic acid 1:1, ChCl/tartaric acid 1:1, ChCl/urea 1:2, ChCl/sorbitol 1:1, ChCl/oxalic acid 1:1) were used as extraction solvents [121]. The highest yields of caffeine and theobromine were achieved when operating at 60 °C for 10 min using 30% water with 500 mg of ground CBS and 10 mL of solvent. Their extraction yields in DESs ranged from 2.145 to 4.682 mg/g for caffeine and from 0.681 to 1.524 mg/g for theobromine. For DES/MAE, the extraction yields of these compounds were 2.502–5.004 mg/g for caffeine and 0.778–1.599 mg/g for theobromine [121]. Determination of the antioxidant activity (DPPH method) showed 24.027–74.805% activity for DES extracts and 11.751–55.444% for DES-MAE extracts [121].

The application of choline-chloride-based DESs for the extraction of coffee and cocoa waste matrices was also carried out by Ruesgas-Ramόn et al. [122]. In this case, the combination of ChCl and lactic acid (2:1 molar ratio) with 10% water was found to be the best choice in terms of the total phenol content detected in cocoa husks (obtaining 52.86 ± 0.78 g GAE/100 g according to the Folin–Ciocalteu method and 0.62 ± 0.08 gGAE/100 g with HPLC) [122]. Interestingly, the extraction yields, when still using the same DES as the solvent, increased when the process was coupled with ultrasound-probe-assisted extraction (3 min/constant duty cycle, 200 W, at 72 °C), even compared to heat-stirring-assisted extraction (HSE) (60 °C for 1 h) [122]. The main bioactive compounds identified in the extracts were theobromine, caffeine and chlorogenic acid [122].

Barbosa et al. proposed PEF-assisted technology, as a pre-treatment, to increase the extraction yield of polyphenols from CBS and coffee silver skin (CS) [123]. They added 50 mL of ethanol at 25 °C to 0.1 g of CBS or CS using different ethanol concentrations in water (from 30 to 70% *v*/*v*). Different time intervals of PEF pre-treatment, numbers of pulses, PEF strengths (from 1.5 to 3 kVcm^−1^ for CBS and from 1.30 to 4.40 kV cm^−1^ for CS) and solid–liquid extraction times (from 30 to 120 min) were applied, as well [123]. The optimal conditions for the recovery of polyphenols from CS were a PEF strength of 1.37 kV/cm for 75 min using 62.67% ethanol, while for CBS, a PEF strength of 1.74 kV/cm for 118.54 min using 39.15% ethanol was optimal [123]. Moreover, this protocol registered around 20% higher recovery yields of polyphenols and methylxanthines compared to conventional extraction [123].

UAE and hydrodynamic cavitation (HC) have also been used to develop alternative green extraction methods from this matrix. Notably, HC reactors are gaining importance to assist conventional solid/liquid plant extraction [124]. HC techniques use high-speed rotating cylinders to create cavitation bubbles inside the matrix. Their collapse significantly increases solid/liquid interactions [124]. The optimized extraction protocol, which lasted 15 min (150 W, 19.9 kHz) at around 40 °C, uses an HC reactor with a ternary water/ethanol/hexane (30:49:21) solvent, giving an extract rich in methylxanthines (32.7 ± 0.12 mg/g of theobromine, 1.76 ± 0.08 mg/g of caffeine) and polyphenols (total phenolic content of 197.4 mg GAE per gram of extract, associated with a radical scavenging activity of 62.0 ± 3.1 μg/mL) [124].

Yusof et al. investigated the application of UAE for the recovery of flavonoids from Malaysian cocoa shell extracts [125]. The study focused on three variables: ethanol concentration (70–90% *v*/*v*), temperature (45–65 °C) and irradiation time (30–60 min). Optimizing the procedure led to the highest total flavonoid content, resulting in 7.47 mg RE/g dried weight, which was detected at 55 °C when using 80% ethanol for 45 min [125].

Rebollo-Hernanz et al. focused on a green aqueous extraction method of phenolic compounds from CBS, using the response surface methodology (RSM) and artificial neural networks (ANNs) to optimize various parameters such as temperature, time, acidity and the solid-to-liquid ratio [126]. The best operating conditions to extract phenolic compounds, phenolic acids, *ortho* diphenols, flavanols, flavonols and prothoantocyanidins and to reach the highest antioxidant activity were found to be water with 0% citric acid, 100 °C, 90 min and a 0.02 g cocoa shell/mL S/L ratio [126]. The experimental results obtained when adopting these conditions matched those predicted by the model [126].

Another novel green extraction process has been recently investigated, obtaining very high yields (up to 100%) of phenolic compounds, theobromine, caffeine, catechin and epicatechin in a single extraction step, reducing the consumption of conventional organic solvents [127]. This requires preliminary hot water extraction (PHWE) (140 °C, 10 CV/h, solvent ratio of 25), followed by anti-solvent (60% ethanol in water)-induced precipitation. Subsequently, the obtained supernatant is used for liquid–liquid extraction (using 40% aqueous citrate buffer/30% aqueous phosphate buffer with a 70/30 phase ratio) with consequent ethanol salting-out [127].

Finally, PHWE (pressurized hot water extraction) was also applied by Pagliari et al. for the extraction of methylxanthines from cocoa by-products (optimal conditions: 15% ethanol at 90 °C for five cycles with a static time of 6 min) and, as a result, 156% and 160% increased efficiencies for theobromine and caffeine, respectively, were obtained in comparison with the UAE technique [128].

**Table 1:** Summary of the reviewed extraction procedures.

Starting Material	Extraction Media	Method	T (°C)	Extraction Time	Ref.
Banana peels	Ethanol 50%	UAE	45 °C	1 h	[50]
	CO_2_ with 5% volume of ethanol	SFE	80 °C	150 min	[51]
	Ethanol 50% (*v*/*v*)	HAE	RT	30 s	[52]
	ChCl–ascorbic acid 1:2 DES	Shaking	RT	20 min	[53]
Kepok banana skin	Water	PEF pre-treatment maceration	RT	2 min (highest TPC) 4 min (highest antioxidant activity)	[54]
Citrus peel	ChCl–LeA–MU 1:1.2:0.8 DES 20% water	UAE	50 °C	25 min	[66]
Mandarin peel	CO_2_ (99.97 *w*/*w*), water/SC-CO_2_ pre-treatment	SFE-SWE	40 °C (pre-treatment) 140–219 °C (extraction)	90 min (pre-treatment) 9–15 min (extraction)	[67]
Lime peel	Ethanol 55%	MAE UAE	Below 60 °C (MAE)	45 s (MAE) 4 min (UAE)	[68]
Kinnow mandarin peel	Methanol 80%	UAE	45 °C	60 min	[69]
Citrus peels	Ethanol 50%	UAE	Below 40 °C	30 min	[70]
Orange peel	CO_2_ with 99% purity, ethanol 75%	SFE-PLE	40 °C (pre-treatment) 65 °C (extraction)	20 min pre-treatment) 20 min (extraction)	[71]
Orange and lime peels	Ethanol 80%	HPE	RT	10 min (orange peel) 3 min (lemon peel)	[72]
Citrus peels	Aqueous enzyme solution (Celluzyme MX) 1.5% (*w*/*w*)	Enzyme-assisted aqueous extraction	50 °C	3 h	[73]
Pineapple skin	Ethanol 50%	MAE	60 °C	20 min	[77]
Pineapple peel, core and crown	ChCl–glycerol 1:2 DES with 10% water	MAE	67 °C (drying temperature)	87 s	[78]
Pineapple peel	Ethanol 50%	MAE		40 min	[79]
Pineapple waste	Ethanol 26%	UAE	37 °C	1 min	[80]
Pineapple peel	Ethanol 58%	UAE	62 °C	30 min	[81]
Pineapple skin	Ethanol 50%	UAE	30 °C	5.92 min	[82]
Pineapple peel	Etanol:solution of 1 mol/L HCl (50:50)	UAE	60 °C	30 min	[85]
Pineapple crown, peel and core	Water	UAE MAE	Solvent temperature below 10 °C (UAE)	20.96 min (UAE) 8.99 min (MAE)	[86]
Pineapple leaves, peel and stem	Tetrabutyl ammonium chloride:imidazole:glycerol(1:1:1)/sodium sulfate NADES	UA-LPME	45 °C	17.5 min	[87]
Pineapple core and skin	Water	Autohydrolysis	200 °C	30 min	[88]
Brewers’ spent grain	NaOH 0.75%	MAE	100 °C	15 min	[101]
	ChCl-Gly (1:2) DES, with 37.46% water	MAE	100 °C	13.30 min	[102]
	Supercritical CO_2_ + 60% ethanol	SFE	40 °C	240 min	[103]
	Ethanol 35%	PLE	155 °C	17 min	[104]
	Ethanol:water (4:1 *v*/*v*)	PEF-assisted extraction	RT	14.5 s	[105]
	Water	UAE	47 °C	30 min	[106]
	NaOH 2%	Basic hydrolysis	120 °C	90 min	[107]
Hot trub	Ethanol 58%	UAE	36 °C	30 min	[109]
Cocoa bean shell	CO_2_ with 99.9% purity, ethanol 99.8%	SFE-PLE	40 °C (SFE pre-treatment) 70 °C (PLE) (extraction)	20 min (SFE pre-treatment) 20 min (PLE) (extraction)	[118]
	Absolute ethanol	PLE	90 °C	50 min	[119]
	Water	SWE	220 °C	75 min	[120]
	ChCl-based DES with 30% water	MAE	60 °C	10 min	[121]
Coffee and cocoa wastes	ChCl-lactic acid 1:2 DES with 10% water	UPAE	72 °C	3 min	[122]
Cocoa bean shell and coffee silver skin	Ethanol 62.67% (coffee silver skin) Ethanol 39.15% (cocoa bean shell)	PEF-assisted extraction	25 °C	75 min (coffee silver skin) 118.54 min (Cocoa bean shell)	[123]
Cocoa bean shell	Water:ethanol:hexane (30:49:21)	UAE and HC	40 °C	15 min	[124]
Malaysian cocoa bean shell	Ethanol 80%	UAE	55 °C	45 min	[125]
Cocoa bean shell (different varieties)	Water	Heat-assisted extraction	100 °C	90 min	[126]
	(1) Water (2) Ethanol 60 wt.% (3) 40 wt.% citrate buffer and a 30 wt.% phosphate buffer (70/30 phase ratio)	(1) PHWE (2) Precipitation (3) Liquid–liquid extraction from precipitation supernatant	140 °C (PHWE)	1 L/h for 0.1 L of solvent (PHWE)	[127]
	Ethanol 15%	PHWE	90 °C	5 cycles with static time 6 min	[128]

UAE, ultrasound-assisted extraction; SFE, supercritical fluid extraction; HAE, homogenizer-assisted extraction; ChCl, choline chloride; DES, deep eutectic solvent; PEF, pulsed electric field; LeA, levulinic acid; MU, methyl urea; SC-CO_2_, supercritical CO_2_; SWE, supercritical water extraction; MAE, microwave-assisted extraction; PLE, pressurized liquid extraction; HPE, high-pressure extraction; NADES, natural deep eutectic solvent; UA-LPME, ultrasound-assisted liquid-phase microextraction; Gly, glycerol; UPAE, ultrasound-probe-assisted extraction; HC, hydrodynamic cavitation; PHWE, pressurized hot water extraction.

## 3. Conclusions

The articles reviewed in this paper were found on well-known databases (Scopus, PubMed) and, as regards the innovative extraction techniques, they date, in almost all cases, to the last five years. These studies, although complete, give us some certainty but also leave other questions open. The advantage of using these methods is certainly evident in terms of saving time and energy, as well as having lower solvent disposal costs. However, it remains difficult to make a reliable comparison between the different extraction methods in order to carry out a concrete assessment of which is the most effective and economically advantageous. To do this, it would be necessary to operate on the same matrix, using all the innovative techniques described in this review and not just design studies that compare one or some of the techniques with a traditional one, as has happened so far.

Food wastes and by-products have great hidden nutritional potential, which will otherwise be lost. This applies both to waste matrices traditionally used as secondary raw materials and exploited, even if only partially, for their potential, as well as to wastes that have not yet become resources for the industry and currently only represent an economic burden. For these reasons, the development and optimization of sustainable techniques for the extraction of bioactive compounds from the most disparate food matrices is gaining importance. Furthermore, it has been demonstrated that some new alternative green techniques not only contribute to reducing pollution and wastage but can also be optimized to improve extraction yields and rates compared to conventional methods. Certainly, this constitutes a sustainable practice from the perspective of the circular economy.
